# Adaptive laboratory evolution of *Escherichia coli* K-12 MG1655 for growth at high hydrostatic pressure

**DOI:** 10.3389/fmicb.2014.00749

**Published:** 2015-01-07

**Authors:** Angeliki Marietou, Alice T. T. Nguyen, Eric E. Allen, Douglas H. Bartlett

**Affiliations:** Marine Biology Research Division, Center for Marine Biotechnology and Biomedicine, Scripps Institution of Oceanography, University of CaliforniaSan Diego, La Jolla, CA, USA

**Keywords:** adaptive laboratory evolution, *Escherichia coli*, high pressure, piezophile, *acp*P

## Abstract

Much of microbial life on Earth grows and reproduces under the elevated hydrostatic pressure conditions that exist in deep-ocean and deep-subsurface environments. In this study adaptive laboratory evolution (ALE) experiments were conducted to investigate the possible modification of the piezosensitive *Escherichia coli* for improved growth at high pressure. After approximately 500 generations of selection, a strain was isolated that acquired the ability to grow at pressure non-permissive for the parental strain. Remarkably, this strain displayed growth properties and changes in the proportion and regulation of unsaturated fatty acids that indicated the acquisition of multiple piezotolerant properties. These changes developed concomitantly with a change in the gene encoding the acyl carrier protein, which is required for fatty acid synthesis.

## Introduction

Elevated hydrostatic pressure promotes reduced system volumes and volume changes of activation in chemical equilibria and rates of reactions, respectively (Meersman and McMillan, 2014). Microorganisms growing preferentially at elevated hydrostatic pressures exist in large portions of Earth's biosphere including deep-sea and deep-subsurface locations (Meersman et al., 2013). Despite the fact that the growth attributes of these piezophiles appear to require relatively little evolutionary change (Prieur et al., 2009), increased hydrostatic pressure exerts pervasive effects on many aspects of cellular function and the adaptations required remain incompletely defined (Lauro et al., 2008). Arguably the best understood adaptation is the need for bacterial piezophiles to maintain a sufficient proportion of unsaturated fatty acids in their membranes as a means of tuning phase, viscosity, and/or ion permeability (Bartlett, 2002; Kawamoto et al., 2011).

*Escherichia coli* is the most studied piezosensitive microorganism with the ability to grow to pressures of up to 50 MPa (Zobell and Cobet, 1963). It has been successfully used as a model organism to investigate the effects of increasing pressure on cell processes and structures. *E. coli* belongs to the class of *Gammaproteobacteria* which includes a large proportion of the characterized piezophiles, while as a foodborne pathogen has been subjected to high pressure pasteurization processes potentially promoting the emergence of piezoresistant strains (Vanlint et al., 2012). High pressure affects many cellular processes in *E. coli*, including replication, transcription, and translation. Previous studies have demonstrated DNA synthesis inhibition at 50 MPa while RNA synthesis was abolished completely at 77 MPa (Yayanos and Pollard, 1969; Welch et al., 1993). Interestingly, a moderate pressure of 30 MPa was sufficient to suppress gene transcription (Sato et al., 1996). Protein synthesis was completely inhibited at 68 MPa; aminoacyl-tRNA binding, ribosome translocation, and ribosome stability have all been implicated as the basis of high pressure impacts on translation (Schwarz and Landau, 1972; Groß and Jaenicke, 1990; Alpas et al., 2003). Cell division was affected at pressures ranging from 20 to 50 MPa resulting in elongation of the cells and a filamentous morphology since cell division is affected prior to cell growth and accumulation of biomass (Zobell and Cobet, 1963). Pressure of 10 MPa and higher affect flagellation by disrupting filament polymerization and flagellum rotation (Meganathan and Marquis, 1973). Other structural changes under high pressure include a compact nucleoid structure (Welch et al., 1993).

High pressure growth of *E. coli* at 30 or 50 MPa induces a cascade of stress responses with the concomitant regulation of a series of genes; such cascade networks include heat and cold stress responses (Welch et al., 1993; Welch and Bartlett, 1998; Aertsen et al., 2004a; Ishii et al., 2005). Interestingly, heat shock pre-treatment improved the pressure resistance of *E. coli* in subsequent high pressure treatment, suggesting that heat shock proteins might play a protective role (Aertsen et al., 2004a). Finally, sub-lethal pressure treatments (100–500 MPa) increased expression of stress sigma factors and genes involved in spontaneous mutations and triggered the SOS and oxidative stress responses (Aertsen et al., 2004b, 2005; Malone et al., 2006). Recently, a genome wide screening approach confirmed the pleiotropic effects of pressure on *E. coli* identifying a series of genes required for growth at high pressure, including genes involved in DNA replication, cell division, cytoskeleton, and cell envelope physiology (Black et al., 2013).

In this report we investigate the feasibility of employing adaptive laboratory evolution (ALE) techniques to drive the phenotype of a model mesophile in the direction of piezophily. ALE is a powerful tool that relies on the Darwinian principles of mutation and selection (Pourmir and Johannes, 2012). In some cases the selections involve a single selective step (Vanlint et al., 2011), in others a few hundred to a few thousand generations (Lee et al., 2011), and in the extreme case of the long-term studies of Lenski and colleagues they may proceed for more than 50,000 generations (Barrick et al., 2009). Examples of ALE experiments selecting for more extremophilic characteristics in mesophiles include increased salt tolerance (Zhou et al., 2013), increased radiation resistance (Harris et al., 2009), the conversion of a mesophile into a facultative thermophile (Blaby et al., 2012) and the selection for survival to exposures of more than 2 gigapascals of pressure (Hauben et al., 1997). It should be noted that resistance to ultra-high pressure does not confer improved growth at elevated pressures (Hauben et al., 1997; Lauro et al., 2008). In this study, following 505 generations of selection we have isolated a strain with the ability to grow at 62 MPa and alter membrane fatty acid composition in response to pressure. A mutation has been identified in a gene involved in lipid metabolism that could contribute in the improved piezotolerance of the newly isolated strain.

## Materials and methods

### Growth conditions

**Figure 1:**
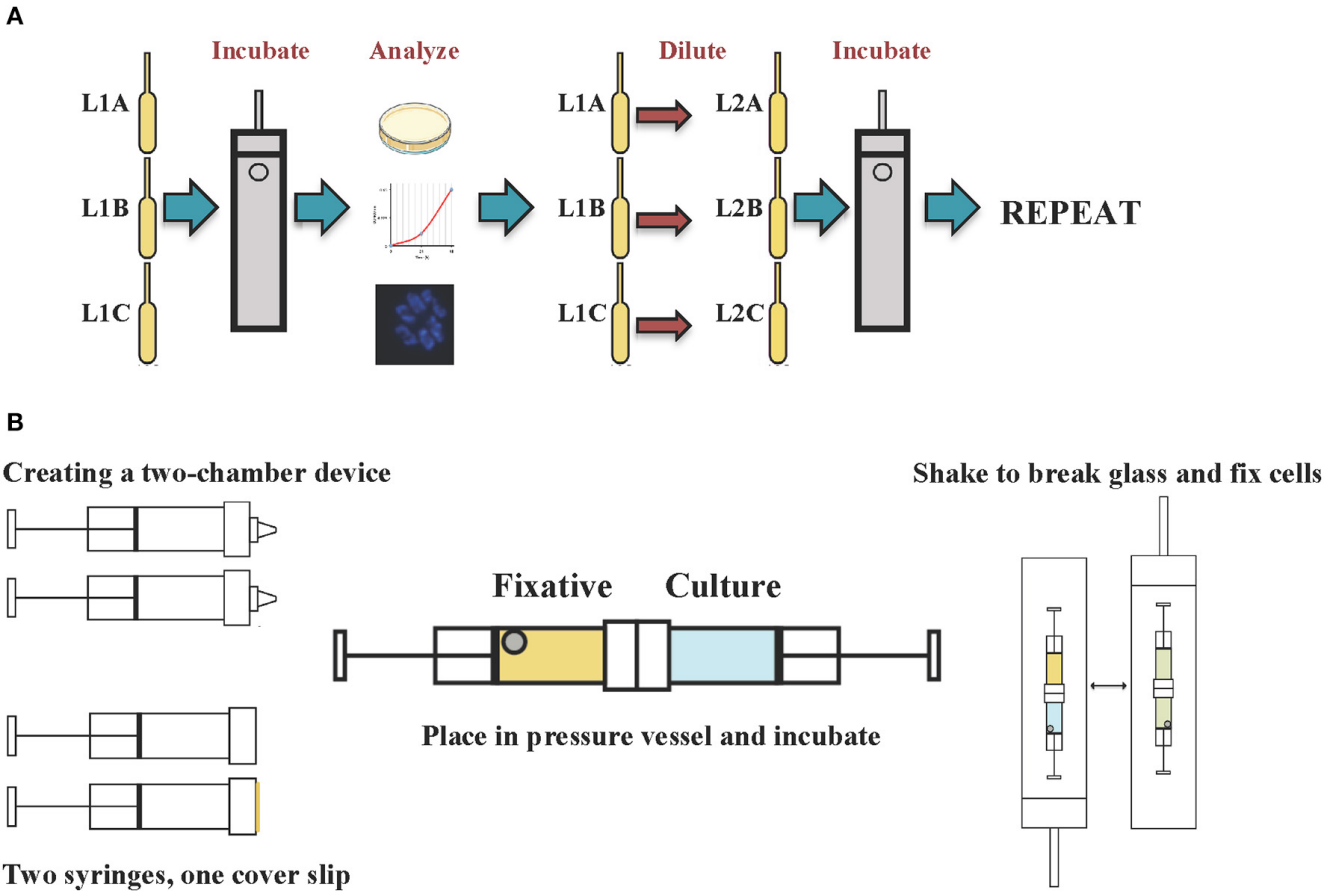
**Schematic diagram of the (A) adaptive laboratory evolution experimental procedure and (B) cell fixation under *in situ* pressure conditions**.

*E. coli* K-12 MG1655 was grown in triplicate lineages (A, B, and C) under fermentative conditions at 37°C using Luria Bertani (LB) medium supplemented with glucose (11 mM) and HEPES buffer (100 mM, pH 7.5) in 5 ml polyethylene transfer pipette bulbs kept within stainless steel pressure vessels (Figure 1A). Initial incubations were performed at 41 megapascal (MPa) and the growth was measured after 48 h of incubation. The cultures were allowed to reach a minimum optical density (OD) at 600 nm of 0.45 (corresponding to a cell density of 4.5 × 10^8^ cells ml^−1^), before the cultures were diluted 1000-fold in fresh medium and reincubated at high pressure. Each triplicate subculture was assigned a new lineage number (i.e., L2A, 2B, 2C). The pressure was gradually increased in increments of 7 MPa when cell yield exceeded 4.5 × 10^8^ cells ml^−1^. Single clonal isolates from each culture were obtained by streaking LB agar plates and incubating for 48 h at 37°C.

### Microscopy and image analysis

Cells were fixed with 2% glutaraldehyde in 0.1 M sodium cacodylate buffer (pH 7.4) under *in situ* pressure conditions (Figure 1B) (Chastain and Yayanos, 1991). Two Sarstedt syringes were joined together using silicone adhesive creating a device with two chambers separated by a circular glass cover slip. The first chamber contained the culture while the second chamber contained the fixative solution and a stainless steel ball. The conjoined syringe was placed in a pressure vessel and incubated at 37°C, at the desired pressure. To fix the cells the pressure vessel was shaken vigorously to cause the steel ball to break the glass cover slip and mix the fixative with the culture at *in situ* pressure conditions.

For epifluorescence microscopy the fixed cells were immobilized onto a 0.2 μm polycarbonate membrane (EMD Millipore) and stained using 4′, 6′ -diamidino-2-phenylindole (DAPI) nucleic acid stain (Vector Laboratories, Inc). The stained samples were viewed at 1000 fold magnification on an Olympus BX51 fluorescence microscope (Olympus). TEM samples were post fixed with 1% osmium and 2% of uranyl acetate and embedded in Ducurpan at 60°C for 36 h. Ultra-thin sections (60 nm) were cut with a diamond knife on Leica Ultracut UCT Microtome and post stained with uranyl acetate and lead. Images were captured on FEI Tecnai spirit at 80 KV.

### Fatty acid analysis

Fatty acid analysis was performed by MIDI Labs (Newark, DE) on frozen cell pellets harvested during the early logarithmic phase of growth. Saponification, methylation, extraction, and base wash were performed before the fatty acid methyl esters were analyzed on an Agilent/HP 6890 gas chromatograph. Using a pattern recognition software, Sherlock MIS, the fatty acid composition of each sample was compared to a stored database. The software was able to identify each component of the analyzed sample, producing a composition report including the relative amount (%) of the named fatty acids.

### DNA purification and genetic analysis

Cells growing at exponential phase were centrifuged at 14,000 × g for 2 min and the cell pellets were used for DNA purification. The Wizard Genomic DNA purification kit (Promega) was used according to the manufacturer's instructions. The purified DNA was used as template for the amplification of the *acpP, fabA, fabB*, and *fabF* genes (see Table S1 for primer sequences and cycling parameters). Each PCR reaction contained 45 μl of Platinum PCR Supermix (Life Technologies), 2 μl of each primer (10 μM) and 1 μl of template DNA. The PCR products were purified using the QIAquick PCR Purification kit (Qiagen) and submitted for direct sequencing by Retrogen Inc. (San Diego, CA) using the respective primers in both directions.

## Results

### *E. coli* growth at high pressure

**Figure 2:**
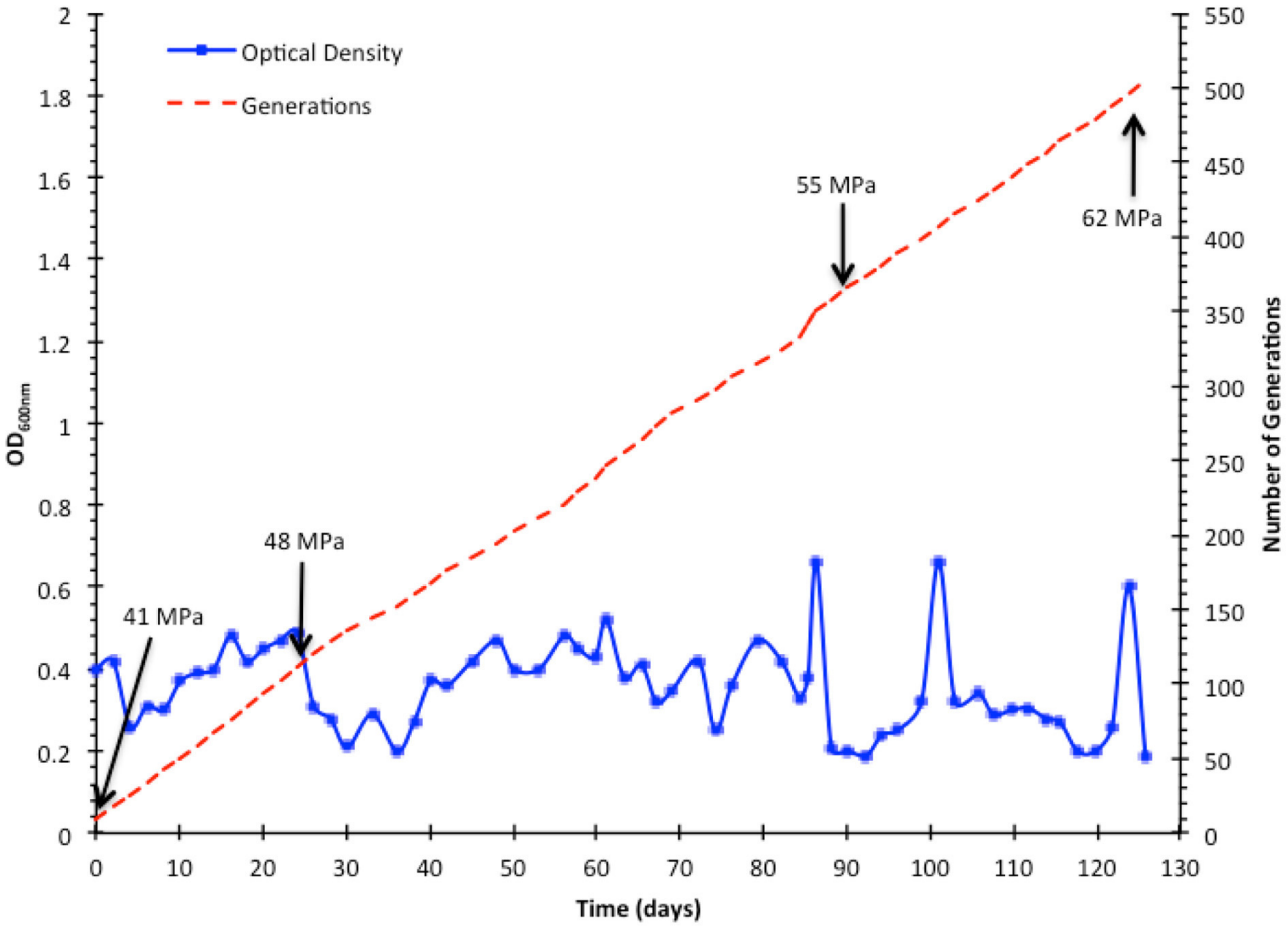
**Directed evolution of *E. coli* K-12 MG1655 to growth at high pressure**. Cultures were grown under fermentative conditions to a minimum OD of 0.45 before they were diluted and incubated at increasing pressure at increments of 7 MPa. The OD values presented were recorded after 48 h of incubation for each subculture for lineage A.

The ALE of *E. coli* for growth at high pressure was initiated by incubating triplicate cultures at 41 MPa. *E. coli* was grown at 41 MPa for 103 generations prior to increasing the pressure to 48 MPa for 247 generations, followed by incubation at 55 MPa for 130 generations, and finally further increased to 62 MPa for 25 generations (Figure 2). The total time required was 126 days and the total number of generations was 505. Improved high pressure growth of isolated strains was not evident until after selection for growth at the last pressure tested, 62 MPa, and this was only evident in cells derived from the lineage A population. Lineage B and C did not respond as well as lineage A to the increasing pressure, failing to produce any cells capable of growth above 55 MPa. The last subculture collected was L62A, and from this population a single clonal isolate, designated strain AN62, was obtained from an LB agar plate.

**Figure 3:**
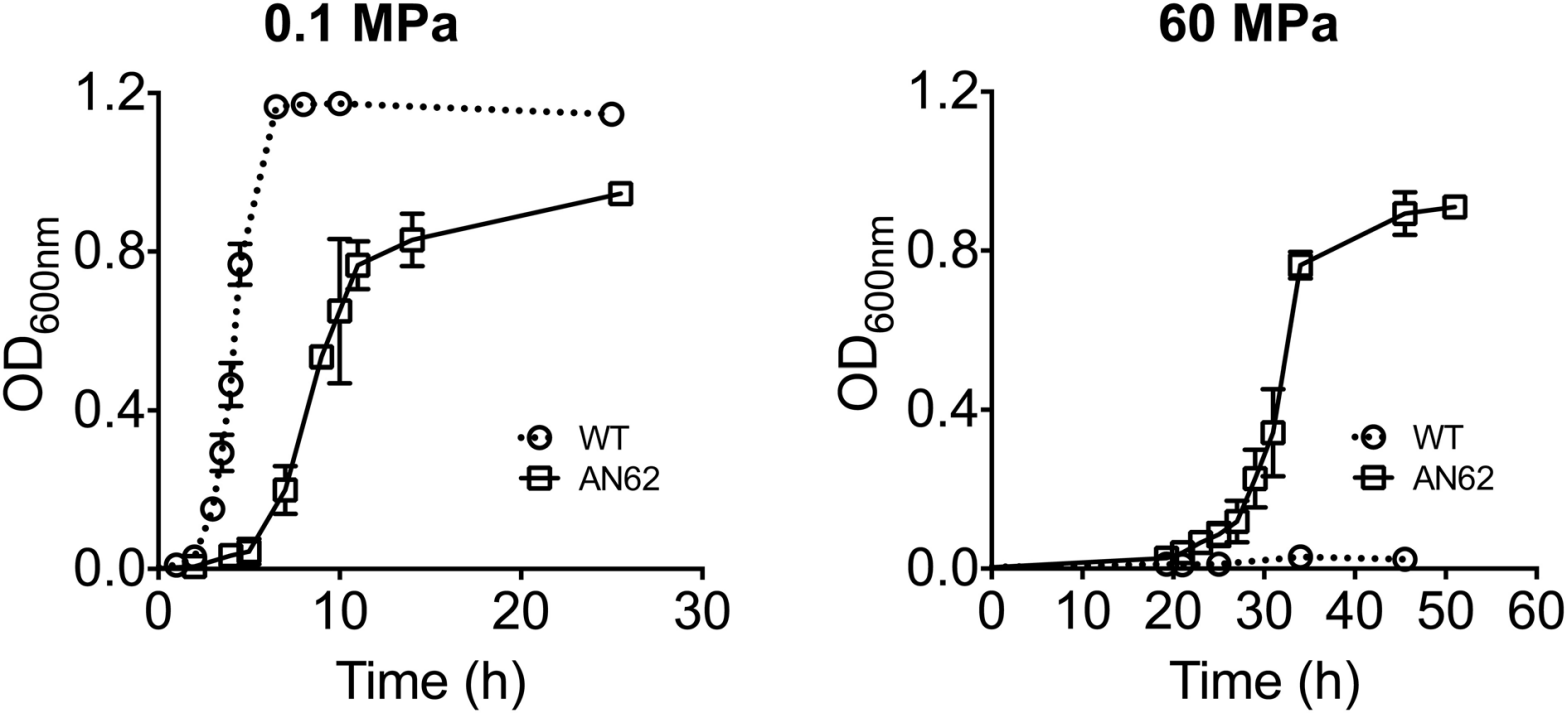
**Growth curves of *E. coli* K-12 MG1655 parental and pressure adapted AN62 strain at atmospheric (0.1 MPa) and high (60 MPa) pressure**. Cultures were grown micro-aerobically in LB supplemented with glucose and HEPES buffer at 37°C. WT, parental strain.

**Figure 4:**
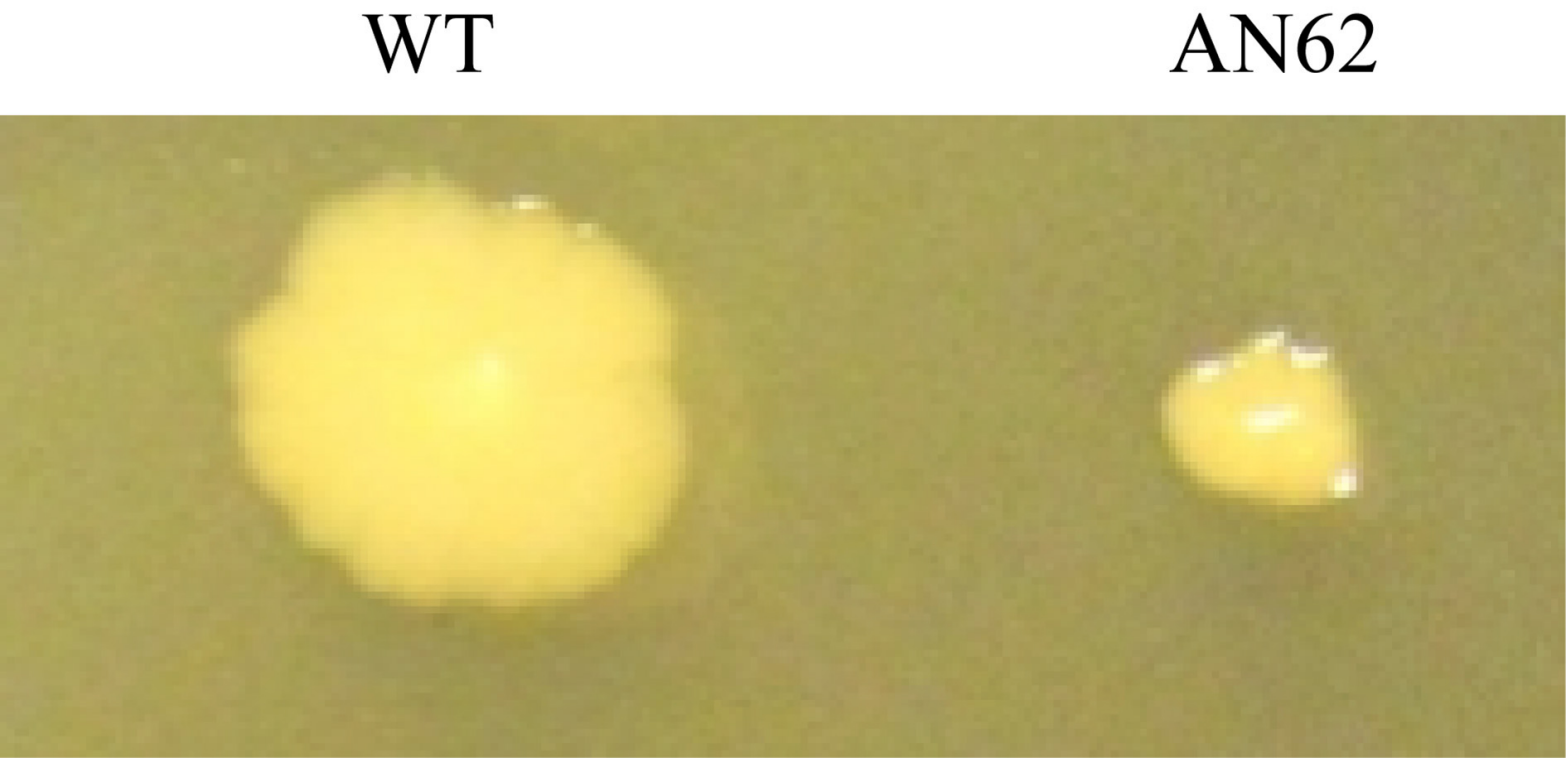
**Colony morphology of *E. coli* parental and AN62 strain**. Cells were plated on LB plates and incubated for 48 h at 37°C at 0.1 MPa. WT, parental strain.

At atmospheric pressure the growth of clonal isolate AN62 was slower than the parental strain in liquid medium (Figure 3). The parental strain had a doubling time of 17 min while AN62 strain had a doubling time of 44 min when grown at 0.1 MPa. In contrast at 60 MPa the parental strain was unable to grow, while AN62 grew after an extended lag phase of ~20 h with a doubling time of 70 min (Figure 3). The same was true for colonial growth on agar plates; AN62 strain formed smaller colonies following a 48-h incubation compared to the parental strain (Figure 4).

### Cell morphology at high pressure

**Figure 5:**
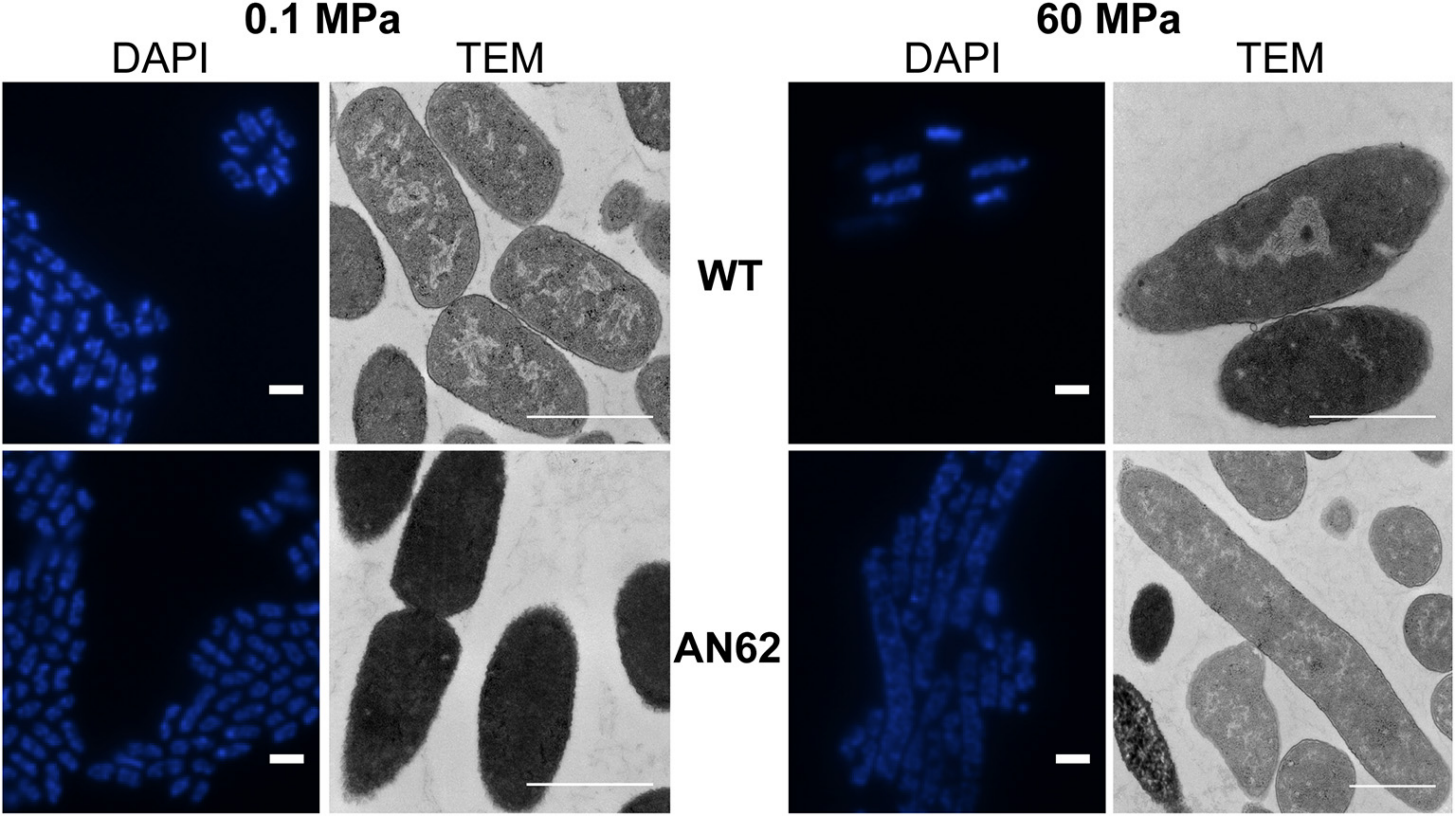
**Epifluorescence and TEM microphotographs of *in situ* fixed parental and AN62 strain cells at atmospheric (0.1 MPa) and high (60 MPa) pressure**. Cultures were grown micro-aerobically in LB supplemented with glucose and HEPES buffer at 37°C. WT, parental strain. Scale bar indicates 1 μm.

Epifluorescence microscopy and transmission electron microscopy (TEM) was used to determine whether any morphological changes accompanied the improved high pressure growth phenotype. The DAPI-stained parental strain (2 ± 0.5 μm) and evolved strain AN62 (2 ± 0.4 μm) exhibited the typical rod shaped morphology of *E. coli* at atmospheric pressure (Figure 5). At 60 MPa the few parental strain cells that were present maintained a rod shaped morphology, while AN62 cells primarily existed as long filaments with an average size of 6.6 ± 2 μm (Figure 5). The parental strain cells appeared longer at 60 MPa (2.5 ± 1 μm, *p* = 0.037) compared to atmospheric pressure.

TEM confirmed the overall morphology indicated by the epifluorescence observations. Interestingly AN62 cells appeared darker at atmospheric pressure compared to the parental strain, suggesting the presence of a dense intracellular matrix (Figure 5). At 60 MPa the majority of the AN62 cells appeared as long filaments (7.2 ± 2 μm). Growth at high pressure caused a change in the distribution of the nucleoid in the parental strain as it appeared condensed in the mid-cell region, while the internal structure of AN62 at high pressure resembled that of the parental strain at atmospheric pressure.

### Membrane fatty acid composition

Because a hallmark feature of all piezophilic bacteria is a high ratio of unsaturated to saturated membrane fatty acids, the fatty acid profiles of the parental and evolved strain were also compared. Under atmospheric pressure growth conditions the ratio of unsaturated to saturated fatty acids was similar for both the parental strain and AN62, although the former produced more palmitoleic acid (16:1 ω 7c) and the latter more cis-vaccenic acid (18:1 ω 7c). Remarkably, at 60 MPa evolved strain AN62 increased its production of cis-vaccenic acid by about 50%, up to more than 30% of the total fatty acid abundance, largely at the expense of the saturated fatty acid palmitic acid (16:0) (Table 1). There was insufficient biomass production at 60 MPa for the parental strain membrane fatty acid analysis.

**Table 1:** **Fatty acid composition of *E. coli* K-12 MG1655 parental and AN62 strain at atmospheric (0.1 MPa) and high (60 MPa) pressure**.

**Fatty Acid**	**WT**	**AN62**	**AN62**
	**0.1 MPa**	**0.1 MPa**	**60 MPa**
	**% of total composition**		
11:0 3OH	0.04	–	–
12:0	6.11	4.60	3.99
12:0 3OH	0.07	0.06	0.08
13:0	0.09	0.10	–
14:0	9.37	6.14	5.54
14:0 3OH	10.76	8.86	8.13
15:0	0.19	0.13	0.10
16:0	27.72	34.06	22.73
16:0 3OH	0.08	0.07	0.09
16:1 ω5c	0.33	0.31	0.84
16:1 ω7c	31.66	20.46	20.72
17:0	0.04	0.05	0.06
17:0 cyclo	2.22	2.93	3.59
18:0	0.14	0.33	0.36
18:1 ω5c	–	0.11	0.25
18:1 ω7c	9.50	20.02	31.15
18:2 ω6,9c	0.04	0.06	–
19:0 iso	0.03	–	–
19:0 cyclo ω8c	0.05	0.21	1.06
Unknown	1.48	1.48	1.25
Sum (%)	99.99	99.98	99.99
UFA/SFA (%)	94.97	90.24	161.32

*Cultures were grown micro-aerobically in LB supplemented with glucose and HEPES buffer at 37°C. Cells were harvested at the early exponential growth phase. WT, parental strain; UFA, unsaturated fatty acids; SFA, saturated fatty acids*.

### Genetic analysis

**Figure 6:**
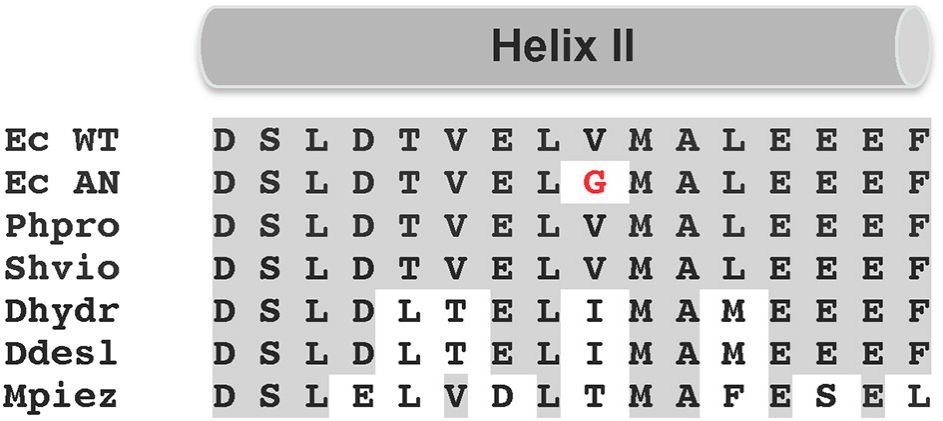
**Multiple sequence alignment of helix II region of various ACP**. Identical residues are shaded gray, while red is used to highlight the glycine in AN62 strain. Ec WT, *E. coli* parental strain; Ec AN, *E. coli* AN62 strain; Phpro, *Photobacterium profundum* SS9; Shvio, *Shewanella violacea* DSS12; Dhydr, *Desulfovibrio hydrothermalis* AM13; Ddesl, *Desulfovibrio desulfuricans* 27774; Mpiez, *Marinitoga piezophila* KA3.

Based on the altered unsaturated fatty acid species abundances produced in AN62 selected genes involved in fatty acid biosynthesis were sequenced. These genes included *acpP*, encoding acyl carrier protein ACP, *fabA*, encoding beta-hydroxydecanoyl thioester dehydrase, *fabB*, encoding beta-ketoacyl-ACP synthase I (KAS I), and *fabF*, encoding beta-ketoacyl-ACP synthase II (KAS II). Only one of these genes was discovered to possess a sequence difference with its homolog in the parental strain. The AN62 *acpP* gene contains a transversion mutation (T to G) at nucleotide position 131 resulting in a valine to glycine (V43G) amino acid change (Figure 6). The evolutionary history of this mutation was followed by assessing its presence or absence in strains derived from past lineages. Based on these analyses it was found that this mutation first appeared in lineage 60A, the same lineage found to possess the first cells adapted for improved high pressure growth (data not shown).

## Discussion

In this study, we used ALE to isolate a novel *E. coli* strain with the ability to grow at pressures that impede parental strain from growing. No *E. coli* strain has ever been documented to grow at pressures exceeding 50 MPa (Ishii et al., 2004). Although AN62 did not exhibit piezophilic growth, which would necessitate improved growth at high pressure compared to atmospheric pressure, its growth characteristics were altered at both ends of the pressure spectrum in a direction toward piezophily. This is the first time any microorganism has had its growth phenotype changed at both low and high pressure, although improved growth at high pressure has been noted in two other studies (Marquis and Bender, 1980; Abe and Horikoshi, 2000).

AN62 strain grew at 60 MPa following an extended lag phase with a doubling time of 70 min (Figure 3). The extended lag phase observed for AN62 at 60 MPa could be attributed to the physiological and molecular changes that the cells have to adjust before exponential growth can proceed at high pressure. Thus, even though AN62 strain has evolved to grow at 60 MPa the changes required to switch from growth at atmospheric pressure to growth at high pressure suggest that the cells required a substantial amount of “fine-tuning,” perhaps reflecting changes in transcription, translation, and/or the activity of key enzymes. Fatty acid biosynthesis is among the processes that are up-regulated during lag phase (Rolfe et al., 2012) and the response of the fatty acid machinery to changes in pressure could have contributed to the observed extended lag phase. Finally, the relatively slow growth rate of AN62 under all pressure conditions indicates that this mutant did not evolve improved growth efficiency but rather improved high pressure growth capacity. The basis of its growth rate reduction, is unknown, but ATP supply and demand is strongly influenced by pressure in piezosensitive bacteria (Marquis, 1982). The significantly slower growth rate of AN62 at 60 MPa could also reflect the constraints imposed on a larger cell volume due to inhibition of cell division at high pressure (Klumpp et al., 2009).

At 60 MPa, AN62 exhibited filamentous morphology in contrast to the parental strain which exhibited only a slightly elongated cell shape. It has previously been reported that *E. coli* assumes a typical filamentous morphology at the non-permissive pressure of 40 MPa (Ishii et al., 2004). Filamentation is a well-documented response of mesophilic bacteria to elevated pressures sufficiently high to inhibit cell division, but not so great as to prevent biomass accumulation (Jannasch, 1987; Yayanos and DeLong, 1987; Kawarai et al., 2004). The basis of this effect may stem from direct effects on the tubulin-like cell division protein FtsZ (Ishii et al., 2004) or through the induction of a DNA damage stress response (Aertsen and Michiels, 2005). When pressures high enough to prevent growth were applied to AN62 (75 MPa), no change in morphology occurred. Thus, along with its growth properties the filamentation response of AN62 was shifted to higher pressures.

Growth at suboptimal pressures also causes substantial changes to the morphology of known piezophiles. *Marinitoga piezophila* KA3 cells are short rods (1–1.5 μm) when grown at the optimal pressure of 40 MPa (Alain et al., 2002). However, when the cells are grown at 10 MPa or atmospheric pressure they appear elongated (Alain et al., 2002). Similarly, *Profundimonas piezophila* cells are rods (4–5 μm) when grown at 60 MPa (pressure optimum 50 MPa) and their morphology changes to 0.8–1.0 μm cocci when grown at pressures lower than 20 MPa (Cao et al., 2014). Interestingly, following nine transfers at atmospheric pressure *M. piezophila* KA3 exhibited the short rod morphotype. These observations highlight the effect of pressure on cell morphology and indicate that adaptation to higher or lower pressures is accompanied by cell size changes.

Strain AN62 produced on overall more cis-vaccenic acid, while its relative amount increased further at 60 MPa. These results indicate that not only had the mutant strain acquired the ability to produce more unsaturated fatty acids, but also it had gained the ability to regulate their abundance in response to the pressure applied. Both of these phenotypes are shared with piezophilic bacteria (Allen and Bartlett, 2000). The molecular mechanisms underlying such a response to increasing pressure remains largely unexplored.

Previous studies highlighted the importance of fatty acid synthesis genes in temperature and pressure response modulation (Garwin and Cronan, 1980; Garwin et al., 1980; Allen and Bartlett, 2000). ACP is a conserved protein that interacts with several other proteins including some with known role in lipid metabolism (Worsham et al., 2003). ACP carries fatty acid synthesis intermediates as thiosters (Rock and Jackowski, 2002). The V43G mutation is located in the helix II region; a site of interaction with several proteins involved in fatty acid synthesis (Jackowski and Rock, 1987). There is a high degree of identity among type II ACP homologs, including position V43 (Jackowski and Rock, 1987). This is also true for most but not all piezophiles. For example, the well-studied piezophiles *Photobacterium profundum* and *Shewanella violacea* possess V43, whereas the piezophiles *Desulfovibrio hydrothermalis* and *M. piezophila* have isoleucine and threonine at position V43, respectively. The presence of V43 in well studied piezophiles is indicating that modification of this residue is not a universal feature of high pressure adaptation.

Previous studies in *E. coli* have established that a V43I substitution affects the ACP equilibrium to a more compact, tightly-folded conformation by stabilizing the hydrophobic core of the protein and decreasing the molecular radius (Keating and Cronan, 1996; Roujeinikova et al., 2002). The V43I substitution is hypothesized to also increase the efficiency of unsaturated fatty acid synthesis (Roujeinikova et al., 2002).

We hypothesize that the mutation uncovered in *acpP* is linked to KAS II activity, both because of the associated increased production of cis-vaccenic acid synthesis observed in AN62 (Garwin et al., 1980; Jackowski and Rock, 1987; Allen and Bartlett, 2000) and because of prior evidence linking ACP structure with KAS II activity (Jackowski and Rock, 1987). KAS II is responsible for increased cis-vaccenic acid production in response to lower growth temperature (Garwin et al., 1980). It catalyzes the elongation of palmitoleic (16:1) to cis-vaccenic acid (18:1). Interestingly, thermal regulation of cis-vaccenic acid synthesis is not controlled at the transcriptional or translation level but is depended on KAS II activity (Garwin and Cronan, 1980). KAS II mutants with impaired activity possessed ACP proteins with the V43I mutation suggesting that V43 residue has an important role in KAS II/ACP interaction with effects on fatty acid synthesis (Jackowski and Rock, 1987).

It is likely that the observed genetic change is necessary but insufficient for piezoadaptation. Additional studies will be needed to identify and confirm all mutations necessary and sufficient for the high pressure phenotypes of AN62. It will also be of interest to determine how much further ALE experiments can drive *E. coli* and other non-piezophiles along the gradient of piezophily. In the case of obligate piezophiles ALE experiments could be used to determine the adaptations required for the evolution of growth and survival at decreased pressures.

ALE could improve our understanding of the evolutionary steps required for the adaptation of life at extremes of pressure. The environmentally relevant exposure of piezosensitive surface microbes to increases in pressure could proceed by a variety of different mechanisms including in association with metazoans undergoing extensive vertical migrations, or in association with large carrion or smaller aggregates of particulate organic carbon. The vertical travel speeds of particle-associated microbes have been estimated to range from 10 to 150 m d^−1^ (McDonnell et al., 2010). At increasing depths travel speed increases by a factor of 10–60%, while pressure increases by 1 MPa every 100 m (Berelson, 2001). Sufficient growth could occur during these transits to enable the acquisition of the requisite number and types of mutations needed for selection of high pressure growth mutants of increased fitness.

Our results demonstrate that it is possible to drive the evolutionary trajectory of a piezosensitive bacterium along the pressure continuum toward piezophily, and to archive the evolutionary history of this process for the subsequent examination of the associated mutational events. ALE experiments performed in this way provide a new approach to characterize the genetic underpinnings enabling microbial life to flourish under the extreme physical constraint of high pressure (low volume change).

### Conflict of interest statement

The authors declare that the research was conducted in the absence of any commercial or financial relationships that could be construed as a potential conflict of interest.
