# Supplementary material for: Adaptive laboratory evolution of Escherichia coli K-12 MG1655 for growth at high hydrostatic pressure
Source: Front Microbiol. 2015 Jan 7;5:749. doi: 10.3389/fmicb.2014.00749 (PMC4285802; doi:10.3389/fmicb.2014.00749)
Supplement: Supplementary file 1 [file Table1.DOCX]

**TABLE S1| Primers and PCR cycling parameters.**

| **Gene** | **Primers (5′ to 3′)** | **PCR Cycling Conditions** |
| --- | --- | --- |
| *acpP* | F 5′-TGAAACTTTGCATGTGAACGGC-3′  R 5′-AATGAAGGCATCCATCTTGCGC-3′ | 95°C, 7 min; for 1 cycle  95°C, 0.5 min; 52°C, 1 min; 72°C, 1 min; for 35 cycles  72°C, 10 min; for 1 cycle |
| *fabA* | F 5′-ACGTGTTAGCTATCCTGCGTGC-3′  R 5′-GCATTGCGGAGGTTTCGCCT-3′ | 94°C, 4 min; for 1 cycle  94°C, 0.5 min; 55°C, 1 min; 72°C, 1 min; for 35 cycles  72°C, 10 min; for 1 cycle |
| *fabB* | F 5′-ATGGCTGATCGGACTTGTTCGGC-3′  R 5′-CCGCATTGGCGCGTAACGTC-3′ | 94°C, 4 min; for 1 cycle  94°C, 0.5 min; 57°C, 1 min; 72°C, 2 min; for 35 cycles  72°C, 10 min; for 1 cycle |
| *fabF* | F 5′-GGTCGTTCGACCGCCTGAGTTT-3′  R 5′-GGCCCGCAAGCGGACCTTTTAT-3′ | 94°C, 4 min; for 1 cycle  94°C, 0.5 min; 57°C, 1 min; 72°C, 2 min; for 35 cycles  72°C, 10 min; for 1 cycle |
